# Complications in Total Joint Arthroplasties

**DOI:** 10.3390/jcm8111891

**Published:** 2019-11-06

**Authors:** Enrique Gómez-Barrena, Eduardo García-Rey

**Affiliations:** Hospital Universitario “La Paz”, Universidad Autónoma de Madrid, 28046 Madrid, Spain; edugrey@yahoo.es

Total joint arthroplasties (TJA) are today considered highly successful operations. Total hip arthroplasty (THA) was the pioneering surgical procedure [1] that predictably modified the clinical and functional outcome of patients affected by degenerative joint disease. “The operation of the century” [2] has evolved through technological and surgical advances, with an eye open to maintaining long-term performance while addressing more demanding functional needs of an aged and not so aged population. Today, patients with joint degeneration seeking treatment may include over 25% of the population aged 65 and above, TJA being the final solution for many of them. However, also in the younger population, demands for more functional recovery of serious joint disorders foster innovation in materials, design and surgical techniques [2]. Success in hip arthroplasties was soon followed by a tremendous development in total knee arthroplasties (TKA), devoted to recover knee motion. In addition, shoulder and elbow arthroplasties were refined and all major joints benefit today from predictable functional recovery following the procedure. Furthermore, it is well known by medical practitioners and the general population that serious articular damage with joint degeneration is today reasonably solved in many cases and that quality of life can be substantially improved in those patients by TJA. 

Costs associated with the burden of TJA are increasing [3], related to the growing demand. However, costs due to productivity loss in younger patients, or decreasing independence in aged patients, may be controlled by these techniques, and savings in societal costs may largely compensate for the procedural costs. Evaluation of resources used in a medical intervention and health related results measuring quality-adjusted life years (QALYs) or health-related quality of life (HRQoL) with time have confirmed the cost-effectiveness and cost-utility of TJA. Both THA and TKA are cost-effective interventions [4], much more consistent than other non-surgical treatments frequently used for advanced primary osteoarthritis, with limited evidence and significant biases. An early TJA performed in an end-stage hip or knee osteoarthritis is far more effective than waiting, under other medical or physical treatments [5].

Well-established joint replacement National Registries have been developed by scientific societies and governments worldwide and have confirmed a prolonged service life of these implants [6], validating not only the techniques but also innovations and long-term population benefits [7]. In addition, they also report the importance of readmissions and complications after TJA, or the fact that 90-day mortality rates are low after THA for osteoarthritis, with different factors, such as surgical approaches, anesthetic procedures or chemical thromboprophylaxis that could explain these rates [8]. In the same way, associated major comorbidities are related to mortality. However, overall mortality has declined in THA and TKA patients despite the fact that the proportion of comorbidities has increased over the past 18 years [9]. Therefore, more complex patients with comorbidities are benefiting from the operations, but potential complications are a concerning consequence.

The number and severity of TJA-related complications remind us of the complexity and seriousness of these surgical procedures. While many patients perform remarkably well without any complications, the risks are often not well-understood by the patient at the time of informed consent. The prognosis of the complicated procedure jeopardizes the expectations of those patients suffering complications. The readmission rate after TJA provides a valid measurement of early complications. This has been associated with comorbidities and is one of the most important reasons for increased costs. Readmission rates 30 or 90 days after surgery range from 5% to 10% [10], most frequently related to infection, wound problems, or cardiovascular complications.

More information from clinical studies is available today about the diagnosis and treatment of joint arthroplasty complications. Both medical and surgical complications may be equally important in altering the patient outcome of TJA. Prophylaxis of complications, early diagnosis and specific treatment may improve this outcome. Therefore, basic research on new technologies or epidemiological, clinical and registry studies on complications and adverse events after TJA related to patient factors (comborbidities, sex, age, smoking) or surgical issues (surgical approach, implant, time, annual volume for both hospital and surgeons) [11,12] are certainly required to help us understand and prevent complications with the intention of improving the already highly successful TJA outcome, except for those patients suffering from undesired events.

The influence of renal function in postoperative complications [13], the potential role of anticoagulant thromboprophylaxis [14], or the effect of smoking in the outcome of TJA [15] are some of the aspects investigated for TJA complications which are addressed in this special issue. Some views from registry studies (re-revision [16], influence of hospital volume [17]) or technical advances to evaluate or decrease complications [18] have their place in this issue. Finally, a major complication of TJA such as infection cannot be underestimated. From the etiology, including multiresistant microorganisms [19,20], to the risk of failure [21] and re-revision associated with surgical techniques, including one-stage or two-stage revision surgery [15,22], there are different research topics of interest that require attention. Diagnostic procedures [23] and consensus definitions of controversial topics such as low grade infection [24] also deserve more consideration in the scientific debate. Taken together, this Special Issue on the topic of TJA complications aims to deepen this research, offering the reader some aspects on selected debates that intend to clarify and further improve the outcome of these unhappy patients who, looking for an improved quality of life, face a difficult situation that may require complex surgical and medical treatments, long hospital stays and, occasionally, ends in limited recovery.
